# Total meat (flesh) supply may be a significant risk factor for cardiovascular diseases worldwide

**DOI:** 10.1002/fsn3.3300

**Published:** 2023-03-17

**Authors:** Wenpeng You, Shuhuan Feng, Frank Donnelly

**Affiliations:** ^1^ Adelaide Nursing School The University of Adelaide Adelaide South Australia Australia; ^2^ Heart and Lung, Royal Adelaide Hospital Adelaide South Australia Australia; ^3^ Adelaide Medical School The University of Adelaide Adelaide South Australia Australia; ^4^ China Organic Food Certification Center Beijing China

**Keywords:** cardiovascular disease, red meat, saturated fat, total meat (flesh of animals), white meat

## Abstract

Consumption of red meat instead of white meat has typically been associated with cardiovascular diseases (CVDs). Reflecting actual diet patterns, this study explored the role of total meat (red + white) in predicting CVD incidence. Data from 217 countries were extracted from United Nations agencies for the analyses in five steps. Bivariate correlations were applied to examine the relationship between total meat and CVD incidence globally and regionally. Partial correlation was applied to identify that total meat was an independent predictor of CVD incidence while socioeconomic status, obesity, and urbanization were statistically constant. Stepwise linear regression was conducted for selecting the significant predictor of CVD incidence. SPSS 28 and Microsoft Excel were used for correlation analyses. Globally, total meat correlated to CVD incidence strongly and significantly in bivariate correlation models. This relationship remained significant in partial correlation when socioeconomic status, obesity, and urbanization were statistically kept constant. Stepwise multiple regression identified that, second to socioeconomic status, total meat was a significant predictor of CVD incidence. Total meat correlated to CVD incidence in different country groupings. However, the correlations between total meat and CVD incidence were significantly stronger in developing countries than in developed countries. Worldwide, total meat (flesh) consumption correlated to CVD incidence independently, but significantly stronger in developing countries than in developed countries. This correlation is worth exploring further in longitudinal cohort studies.

## Graphical abstract

Reflecting actual diet patterns, this study explored the role of total meat in predicting cardiovascular disease (CVD) incidence worldwide. Further data analyses showed that total meat (flesh) consumption correlated to CVD incidence independently but was significantly stronger in developing countries than in developed countries.

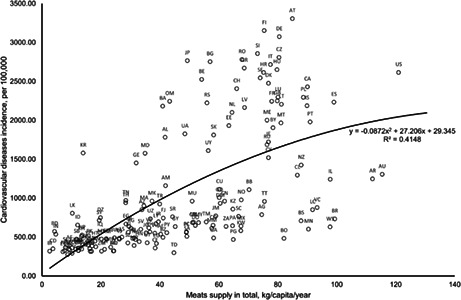

## 1 INTRODUCTION

Cardiovascular diseases (CVDs) are a group of disorders of the heart and blood vessels (WHO, 2021). They represent a major health problem worldwide as the leading cause of death globally. The World Health Organization (WHO) estimated that, representing 32% of all global deaths, around 17.9 million people died from CVDs in 2019 (WHO, 2021).

A complete understanding of the etiology of CVDs remains elusive to the public and professionals. However, studies have revealed that most CVDs are preventable by addressing behavioral risk factors such as unhealthy diet, obesity, and physical inactivity (WHO, 2021). A high intake of saturated fat has been associated with increased levels of low‐density lipoprotein cholesterol (WHO, 2021). It is generally accepted that, due to saturated fat, meat consumption contributes to the development of CVDs as plaques form in people's blood vessels (Malakar et al., 2019; Varghese & Kumar, 2019). This is important as red meats typically have more saturated fat than white (poultry) meats. In the last few decades, a large number of cohort and case–control studies have controversially and circumstantially reported that red meat may be a detrimental diet component for developing CVDs (Astrup et al., 2020; Papier et al., 2021). However, none of these studies revealed that there are significantly different levels of cholesterol content between red meat and white meat (Beauchesne‐Rondeau et al., 2003; Bergeron et al., 2019; Davidson et al., 1999; Mateo‐Gallego et al., 2012; Melanson et al., 2003; Scott et al., 1994). Complementarily, a recent study revealed that choosing white meat over red meat does not necessarily reduce the undesirable type of cholesterol or decrease the risk of CVDs (Bergeron et al., 2019).

Singling out red meat for advancing its effects on increasing CVD risk may also have one or more following biases:

1. It has been suggested that there was no substantial difference between “red meat” and “white meat” in terms of the nutrient components (Murphy et al., 2014; You & Henneberg, 2016a). It is well known that saturated fat in red meat is the primary culprit for increasing the risk for CVD development (Briggs et al., 2017; Nettleton et al., 2017). However, although less than in red meat saturated fats also exist in white meat (American Heart Association, 2023; Damigou et al., 2022; Duarte et al., 2019; Food and Agriculture Organization [FAO], 2023; USDA, 2019).
2. A large majority of people consume diets that are a combination of red meat and white meat. The separation of meats, however, is not reflected in a range of study purposes. This may have created a bias in previous studies focusing on an intake of red meat, instead of total meat, as a risk factor for CVDs (Al‐Shaar et al., 2020).
3. In the past based on observational studies associating red meat intake and CVD risk, dietary recommendations have been made to the public that less red meat and more white meat should be consumed. These recommendations are widespread and can be found in students' textbooks, hospital healthy diet pamphlets, and even in government dietary guidelines, such as United States Department of Agriculture (USDA) (Oppenheimer & Benrubi, 2014). However, the effects of the recommendations are worthy of further analysis as CVD incidence keeps rising worldwide.

In consideration of the above biases that occurred in previous research, we ascertain that total meat (flesh of animals) may be a significant risk factor for CVDs. This hypothesis was examined through analysis of the latest available population‐level data published by several United Nations (UN) agencies. Socioeconomic status, urban living, and obesity were considered confounding factors. This study also identified that, secondary to socioeconomic status, total meat is another significant risk factor for CVDs.

## 2 MATERIALS AND METHODS

### 2.1 Data collection and selection

The population‐specific data published by the agencies of the United Nations were collected for this ecological study. We simply extracted a whole country list comprising 217 countries from the World Bank website, and then all the variables were matched within this list. As per the World Bank, the country in this study only refers to the geographic territory or region for which authorities report separate datasets on health, demography, and economic situation, but does not necessarily mean political independence (The World Bank, 2022). The terms country and population are interchangeably used to represent the geographic region or territory in this study.

The independent or predicting variable, total meat supply per capita in 2017 was sourced from the FAOSTAT Food Balance Sheet (FBS; FAO, 2017). It is expressed with kg/capita/year which measures the average amount of “flesh of animals used for food” supplied to each individual in 2017. The total meat supplied to the population includes both red meat and white meat, which are the animal flesh from cattle (beef and veal), buffalo, pig, sheep (mutton and lamb), goat, horse, chicken, goose, duck, turkey, rabbit, game, and offal (FAO, 2017).

The dependent variable, CVD incidence rate (new cases per 100,000 population) for all ages in 2017 was sourced from the Institute for Health Metrics and Evaluation (IHME, 2020). Based at the University of Washington, IHME is an independent and reputable research institute focusing on the area of global health statistics. Not only does it provide data concerning the most pressing global health challenges, but it also offers strategies for addressing health through research.

As CVD is a consequence of multiple etiologies, to rule out the confounding effects on the relationship between total meat and CVD incidence, we extracted the following variables which have been associated with CVD incidence:

1. Socioeconomic status which is measured with per capita GDP PPP (gross domestic product converted to international dollars using purchasing power parity rates) in 2014 was downloaded from the World Bank databank (The World Bank, 2018). Socioeconomic status is associated with the increase in life span, education, and acquisition of lifestyle‐related risk factors, such as obesity and diabetes (Gaziano et al., 2010). It also determines the magnitude of early detection of CVDs.
2. Urbanization measuring the percentage of the population living in urban areas in 2014 was sourced from the World Bank (The World Bank, 2018). Urban living imposes lifestyle limitations and impacting personal choice which impacts people's opportunities to be cardiovascularly healthy (Smith et al., 2022). Accompanying the process of modernization and industrialization, urbanization has been associated with a human lifestyle change, such as more meat intake (You & Henneberg, 2016a, 2016b), cheap and easily available foods high in salt, sugar, and fats (Smith et al., 2022), and less physical exercise (Allender et al., 2008).
3. The obesity prevalence rate indicating the percentage of the population aged 18+ with body mass index (BMI) ≥ 30 kg/m^2^ in 2014 was extracted from the database of the WHO Global Health Observatory (2022). Obesity is a multifactorial health challenge that contributes directly to the incidence of CVD. Other risk factors include hypertension, type 2 diabetes, dyslipidemia, and atherosclerosis in both adults and children (Powell‐Wiley et al., 2021; Schwartz et al., 2017; Unamuno et al., 2018). Furthermore, in a number of studies, total meat intake has been extensively associated with obesity which may have been contributing to CVD incidence (Livingstone & McNaughton, 2016; You & Henneberg, 2016a, 2016b).

All variables were downloaded and saved in Microsoft Excel® 2016 for analysis. In this study, each country or population was considered as an individual research subject in our data analysis. The number of countries included for different variables may differ somewhat because not all information on other variables was uniformly available for all countries due to unavailability from relevant United Nations agencies.

### 2.2 Statistical analyses

With reference to the previous studies (You et al., 2020, 2022; You & Donnelly, 2022; You & Henneberg, 2018, 2022; You, Henneberg, et al., 2022), the relationship between total meat and CVD incidence was examined in five steps:

1. Scatter plots were produced with the original data in Microsoft Excel® 2016 to explore and visualize the strength, shape, and direction of the association between total meat and CVD incidence at the global level. This allowed us to identify if there were outliers in the data set as well.

For the data analysis in SPSS (Steps 2–5), the original data were log‐transformed (natural logarithms) to bring their distributions closer to normal, which may increase the homoscedasticity of data distributions.

2 Bivariate correlations (Pearson's and Nonparametric) were performed for evaluating the strength and direction of the associations between all variables (total meat, CVD incidence, GDP PPP, obesity, and urbanization).
3 Partial correlation of Pearson moment–product approach was conducted to examine the relationship between total meat and CVD incidence while incorporating GDP PPP, obesity, and urbanization as the confounding factors.
4 Standard multiple linear regression (enter) was performed to describe the relationship between the dependent variable (CVD incidence) and the predicting and confounding variables. In order to explore if and how much total meat can statistically explain the individual relationships between total meat and GDP PPP, obesity, and urbanization, the enter multiple linear regression was performed to calculate the correlations between total meat and the confounding variables when total meat was “added” and “not added” as a predicting variable, respectively.
  Subsequently, standard multiple linear regression (stepwise) was performed to select the most significant predicting variable(s) for CVDs when total meat was “added” and “not added” as a predicting variable, respectively.
5 Bivariate correlations (Pearson's *r* and nonparametric) were calculated to investigate the regional correlations between total meat and CVD incidence. For exploring the regional correlations, the countries were grouped for the correlation analyses:
1 the World Bank income classifications: high income, upper middle income, low–middle income, and low income;

In response to the WHO's statement that at least 75% of CVD deaths occur in low‐ and middle‐income countries (LMIC; WHO, 2021). The high‐income countries were singled out for creating a country grouping, and at the same time the LMIC were combined for creating another country grouping. The bivariate correlations were explored between total meat and CVD incidence in both country groupings and then Fisher's *r*‐to‐*z* transformation was applied for comparing the correlations.

2 the United Nations' common practice on defining the developed and developing countries (United Nations Statistics Division, 2016);

As a further response to the above WHO statement, Fisher's *r*‐to‐*z* transformation was applied to compare the correlation coefficients between total meat and CVD incidence in developed countries and in developing countries.

3 the WHO regional classifications: Africa (AFR), Americas (AMR), Eastern Mediterranean (EMR), Europe (EU), South‐East Asia (SEAR), and Western Pacific (WPR) (WHO, 2018);
4 countries with a strong contrast in terms of geographic distributions, per capita GDP levels and/or cultural backgrounds. We analyzed the correlations in the eight country groupings: Asia Cooperation Dialogue (ACD, 2018); the Asia‐Pacific Economic Cooperation (APEC, 2015); the Arab World (The World Bank, 2015), countries with English as the official language (government websites), Latin America (The United Nations Educational Scientific and Cultural Organization, 2014), Latin America and the Caribbean (LAC) (The United Nations Educational Scientific and Cultural Organization, 2014), the Organization for Economic Co‐operation and Development (OECD, 2015) and Southern African Development Community (SADC, 2015).

SPSS v. 28 (SPSS, Inc.) and Microsoft Excel 2016® were used for our data analyses. The significance was kept at the .05 level, but .01 and .001 levels were also reported in the data analyses. Stepwise multiple linear regression analysis criteria were set at the probability of *F* to enter ≤.05 and probability of *F* to remove ≥.10.

## 3 RESULTS

**FIGURE 1:**
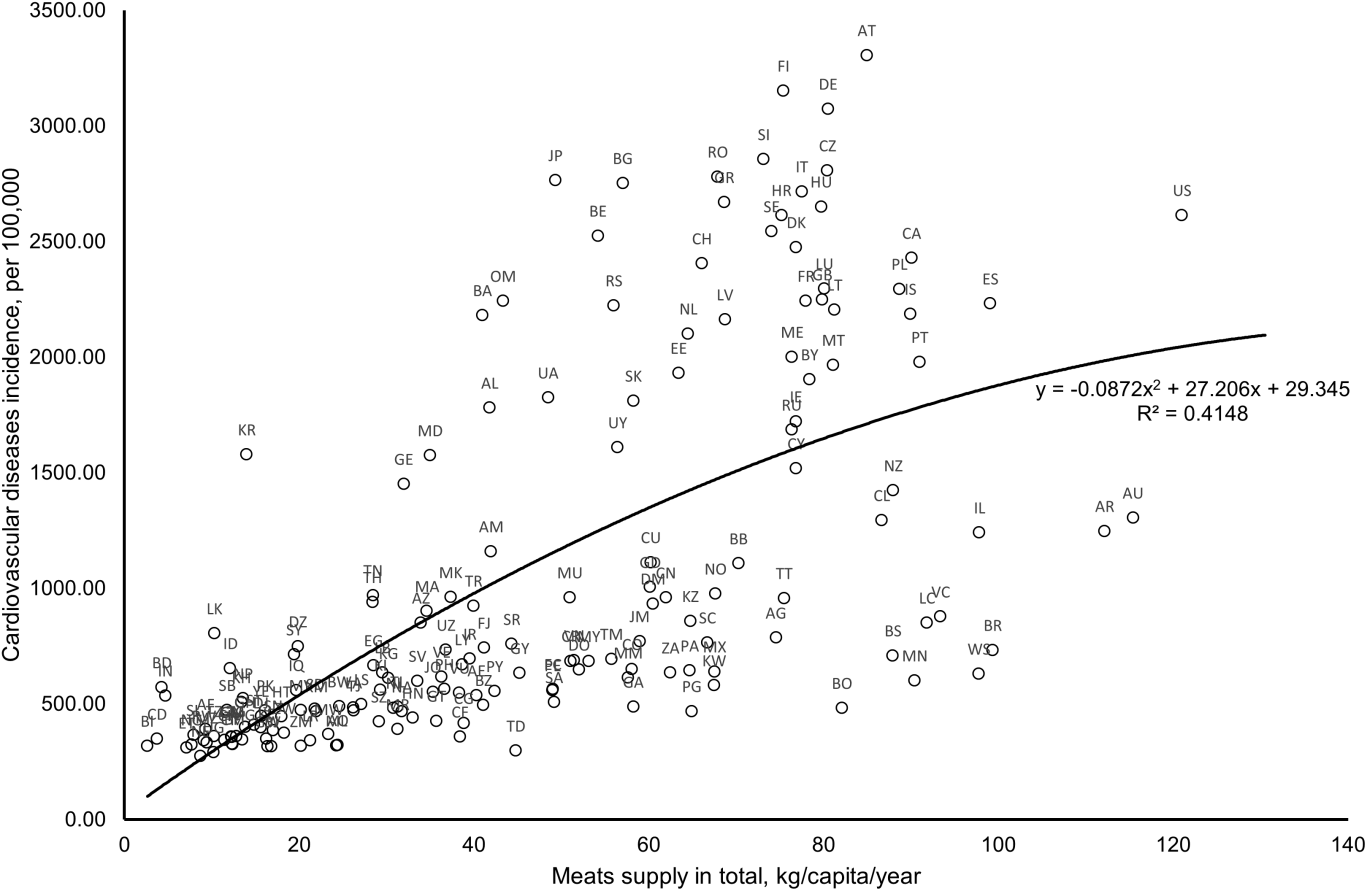
Polynomial correlation plot of total meat and cardiovascular disease incidence. (Data source and definition: Total meat, measured with total meats supply quantity (kg/capita/year) (Food and Agriculture Organization, 2017); Cardiovascular disease incidence (per 100,000) sourced from the Institute for Health Metrics and Evaluation. Both variables were not log‐transformed for correlation analysis.)

Figure 1 shows the unadjusted correlation between total meat and CVD incidence rate. The relationship was noted to be best described by a polynomial equation (*y* = −0.0872*x* ^2^ + 27.206*x* + 29.345) with moderately strong correlation (*r* = .644, *p* < .001). There seems to be no outlier observed in the plots (Figure 1).

Pearson correlation and nonparametric analyses revealed that total meat was significant and strongly correlated to CVD incidence (*r* = .688 and *r* = .752, *p* < .001, respectively) (Table 1). The moderate to strong, but significant correlations were also observed between CVD incidence and GDP PPP, obesity, and urbanization, respectively. This warranted our selection to include them as the confounding factors in exploring the correlation between total meat and CVD incidence.

**TABLE 1:** Pearson *r* (above diagonal, shaded) and nonparametric (below diagonal) correlation matrix between all variables.

	Total meat	Cardiovascular disease incidence	GDP PPP	Obesity prevalence	Urbanization
Total meat	1	.688**	.738**	.644**	.544**
Cardiovascular disease incidence	.752**	1	.734**	.428**	.526**
GDP PPP	.767**	.775**	1	.502**	.716**
Obesity prevalence	.574**	.473**	.483**	1	.546**
Urbanization	.563**	.555**	.752**	.584**	1

*Note*: Data source and definition: Total meat, measured with total meats supply quantity (kg/capita/year) (FAO, 2017); Cardiovascular disease incidence, the number of new cases per 100,000 (IHME, 2020); Per capita GDP PPP, measured with the per capita purchasing power parity (PPP) value of all final goods and services produced within a territory in a given year (The World Bank, 2014a); Urbanization, measured with the percentage of population living in urban area (The World Bank, 2014b); Obesity prevalence, measured with the percentage of population aged 18+ with body mass index equal to or over 30 kg/m^2^ (The World Health Organization, 2022). All the data were log‐transformed for correlation analysis. Significance levels: **p < .01. Number of country range, 168–193.

Partial correlation analysis revealed that total meat was still a significant predictor of CVD incidence while GDP PPP, obesity, and urbanization were statistically kept constant (*r* = .308, *p* < .001, Table 2). When meat intake was statistically stabilized as a confounding factor in partial correlation analysis, it was revealed that: (1) GDP PPP and urbanization were identified as significant independent predictors of CVD incidence (*r* = .463 and *r* = .249, *p* < .001, respectively). (2) Obesity showed nearly nil correlation to CVD incidence (Table 2).

**TABLE 2:** Partial correlations between CVD incidence and independent variable when total meat was included as the independent and confounder, respectively.

Variables	Partial correlation to CVDs			Partial correlation to CVDs		
	*r*	*p*	df	*r*	*p*	df
Total meat	.308	<.001	163	‐	‐	‐
GDP PPP	‐	‐	‐	.463	<.001	168
Obesity prevalence	‐	‐	‐	−.029	.714	165
Urbanization	‐	‐	‐	.249	<.001	168

*Note*: Data source and definition: Total meats, measured with total meats supply quantity (kg/capita/year) (FAO, 2017); Cardiovascular disease (CVD) incidence, the number of new cases per 100,000 (IHME, 2020); Per capita GDP PPP, measured with the per capita purchasing power parity (PPP) value of all final goods and services produced within a territory in a given year (The World Bank, 2014); Urbanization, measured with the percentage of population living in urban area (The World Bank, 2014); Obesity prevalence, measured with the percentage of population aged 18+ with body mass index equal to or over 30 kg/m^2^ (The World Health Organization, 2014). All the data were log‐transformed for correlation analysis. “‐” Included as the confounding factor.

Standard multiple linear regression (enter) analysis was applied to further predict CVD incidence with total meat intake, GDP PPP, obesity prevalence, and urbanization considered as the predicting variables. When total meat was “not added” as one of the predicting variables, GDP PPP was the only significant predictor of CVD incidence (*β* = .751, *p* < .001, Table 3A). While total meat was entered as a predicting variable, it showed a significant correlation to CVD incidence together with GDP PPP (*β* = .282 and .673, *p* < .001, respectively, Table 3B).

**TABLE 3:** Results of multiple linear regression analyses to demonstrate how the variables competed each for predicting CVD incidence.

(A) Enter multiple regression to sort significant predictors of cardiovascular disease incidence				
	Total meats not added		Total meats added	
Variables entered	*β*	Sig.	*β*	Sig.
Total meats	Not added	Not applicable	.282	<.001
GDP PPP	.751	<.001	.673	<.001
Obesity prevalence	.053	.404	−.108	.108
Urbanization	−.050	.532	−.032	.656

(B) Stepwise multiple regression to sort significant predictors of cardiovascular disease incidence					
Rank	Total meats not added		Rank	Total meats added	
	Variables entered	Adjusted *R* ^2^		Variables entered	Adjusted *R* ^2^
1	GDP PPP	.548	1	GDP PPP	.627
2	Obesity prevalence	Nonsignificant predictor	2	Total meats supply	.648
3	Urbanization	Nonsignificant predictor	3	Obesity prevalence	Nonsignificant predictor
4	Total meats supply	Not added	4	Urbanization	Nonsignificant predictor

*Note*: Stepwise multiple linear regression modeling is reported. The contribution of variables is listed in order of how much they contribute to cardiovascular disease (CVD) incidence. Data source and definition: Total meat, measured with total meats supply quantity (kg/capita/year) (Food and Agriculture Organization, 2017); Cardiovascular disease incidence, the number of new cases per 100,000 (IHME, 2020); Per capita GDP PPP, measured with the per capita purchasing power parity (PPP) value of all final goods and services produced within a territory in a given year (The World Bank, 2014); Urbanization, measured with the percentage of population living in urban area (The World Bank, 2014); Obesity prevalence, measured with the percentage of population aged 18+ with body mass index equal to or over 30 kg/m^2^ (The World Health Organization, 2014). All the data were log‐transformed for correlation analysis.

In the standard multiple linear regression (stepwise) analysis, when meat intake was not entered as a CVD incidence predictor, GDP PPP was the only significant predictor for CVD incidence (*R* ^2^ = .548, Table 3B). When meat intake was incorporated as an independent variable, second to GDP PPP, it was placed as a major predictor for CVD incidence with increasing *R* ^2^ to .648 (Table 3B). Obesity and urbanization were not identified as the major predictors of CVD incidence.

Table 4 shows the bivariate correlations between total meat and CVD incidence in different country groupings. In general, total meat intake was in positive correlation to CVD incidence in each country grouping. The strength and significance level may depend on the sample size and the role of total meat in predicting CVD incidence. Fisher's *r*‐to‐*z* transformation identified that total meat was insignificantly stronger and correlated to CVD incidence in low‐ and middle‐income countries than in high‐income countries (*z* = 2.52, *p* < .010, and *z* = 3.49, *p* < .001 in Pearson's r and nonparametric models, respectively). Similarly, the correlation in the developing country grouping had a significantly stronger correlation than in the developed country grouping (*z* = 2.65, *p* < .010, and *z* = 3.76, *p* < .001 in Pearson's *r* and nonparametric models, respectively). These results may suggest that the role of total meat was significantly stronger in low‐ and middle‐income countries than in high‐income countries (Table 4).

**TABLE 4:** Total meat correlated to cardiovascular disease incidence in different country groupings.

Country groupings	Pearson's *r*	*p*	Nonparametric	*p*	Mean of total meat (kg/capita/year)
Worldwide (*n* = 171)	.688**	<.001	0.752**	<.001	49.10
United Nations common practice					
Developed, *n* = 43	.229	.140	0.129	.411	73.95
Developing, *n* = 128	.614**	<.001	0.671**	<.001	39.09
Fisher's *r*‐to‐*z* transformation	Developing vs. developed: *z* = 2.65, *p* < .010		Developing vs. developed: *z* = 3.76, *p* < .001		
World Bank income classifications					
High income (HI), *n* = 50	.211	.141	0.209	.144	75.84
Low income (LI), *n* = 25	.307	.135	0.377	.063	19.57
Low–middle income (LMI), *n* = 46	.385**	<.010	0.476**	<.001	27.36
Upper middle income (UMI), *n* = 50	.332*	.019	0.377**	.007	51.91
Low‐ and middle‐income countries (LI, LMI, UMI), *n* = 121	.597**	<.001	0.672**	<.001	24.58
Fisher's *r*‐to‐*z* transformation	Low and middle income vs. high: *z* = 2.52, *p* < .010		Low and middle income vs. high: *z* = 3.49, *p* < .001		
WHO regions					
African region, *n* = 43	.229	.140	0.129	.411	22.02
American region, *n* = 34	.672**	<.001	0.723**	<.001	63.33
Eastern Mediterranean region, *n* = 18	.472*	<.050	0.486*	<.050	30.69
European region, *n* = 50	.665**	<.001	0.519**	<.001	65.74
South‐East Asian Region, *n* = 9	.108	.781	−0.100	.798	25.54
Western Pacific Region, *n* = 17	.240	.354	0.368	.147	61.00
Countries grouped with various factors					
Asia Cooperation Dialogue, *n* = 28	.300	.121	0.408*	<.050	37.62
Asia‐Pacific Economic Cooperation, *n* = 17	.322	.207	0.424	.090	66.47
Arab World, *n* = 18	.311	.210	0.232	.354	31.14
English as official language, *n* = 46	.789**	<.001	0.865**	<.001	47.29
Latin America, *n* = 20	.608**	<.010	0.704**	<.001	55.90
Latin America and the Caribbean, *n* = 32	.626**	<.001	0.680**	<.001	60.78
Organization for Economic Cooperation and Development, *n* = 37	.303	.068	0.135	.425	76.75
Southern African Development Community, *n* = 16	.790**	<.001	0.768**	<.001	29.57

*Note*: Pearson *r* and nonparametric correlations within country groupings were reported. Data source and definition: Total meat, measured with total meats supply quantity (kg/capita/year) in 2017, the Food and Agriculture Organization; Cardiovascular disease incidence, the number of new cases per 100,000 in 2017, the Institute for Health Metrics and Evaluation. All the data were log‐transformed for correlation analysis.

*p < .05; **p < .01; ***p < .001.

## 4 DISCUSSION

CVDs have been a growing public health concern owing to multiple etiologies including eating red meat. By assessing the relationship between total meat and CVD incidence, this study suggests that total meat may be another significant risk factor for developing CVDs.

A large body of studies have consistently associated meat consumption with individual CVDs. However, almost all of these studies only focused on the role of red meat, instead of total meat or white meat in contributing to CVDs. Compared to white meat, red meat contains more saturated fat, which elevates levels of cholesterol and other atherogenic lipoproteins that contribute to CVDs (Al‐Shaar et al., 2020; Medeiros et al., 2019; Papier et al., 2021; Wang et al., 2022). In the past years, red meat‐associated metabolites, such as trimethylamine‐*N*‐oxide (TMAO), have gained attention in their relationship between red meat eating and CVDs (Wang et al., 2022). Similarly, l‐carnitine, the active form of dietary carnitine and a driver of TMAO production in red meat, has been targeted by researchers for exploring CVD prevention and treatment (Wang et al., 2022; Zhao et al., 2022).

A limited number of studies have explored the role of total meat in contributing to CVDs. Four studies involving a total of 213,722 participants identified that total meat consumption was correlated to 9%–28% increase in the risk of stroke (Bernstein et al., 2012; Kim et al., 2017; Larsson et al., 2011a, 2011b). A meta‐analysis of prospective cohort studies conducted by Kim et al. (2017) revealed that greater consumption of total meat may lead to an 18% increase in the risk of stroke. Bergeron et al. (2019) suggested that limiting total meat consumption (whether red or white) may reduce cholesterol levels leading to lower CVD risk. Similar results were revealed in a prospective cohort study of men across 30 years that greater intake of total meat may be a risk factor for coronary heart defect (Al‐Shaar et al., 2020). While such studies support our hypothesis, it is noted that the results may be more circumstantial as these studies were conducted within particular populations.

Only a relatively few studies have looked at the relationship between white meat consumption and CVDs. The study findings were controversial (Abete et al., 2014; Kim et al., 2017; Zhong et al., 2020). Kim et al. (2017) identified that white meat consumption was associated with a 13% increased risk of stroke. A meta‐analysis concluded that white meat protected against CVDs risk and suggested that white meat could be a healthier alternative to red meat and processed meat consumption (Lupoli et al., 2021). Contradicting this suggestion, a randomized controlled trial revealed that red meat and white meat both elevated cholesterol to an identical level in healthy men and women when saturated fat levels were equivalent in two meat subgroups (Bergeron et al., 2019). Such contradictory findings argue for further study of the impact of total meat consumption.

It is worth analyzing the underlying reason for the differences between red and white meat consumption and their correlation to the adverse effects of CVD. As the other meat subgroup, white meat represents a minor portion of the human diet (FAO, 2017). Although it also contains CVD‐associated substances (saturated fat, l‐carnitine, TMAO, sodium), in comparison with red meat, the levels are very low (Murphy et al., 2014; Wang et al., 2019; You & Henneberg, 2016a). For instance, generally speaking, white meat only contains a quarter of carnitine in red meat (Spence et al., 2021). This means the impact of these substances in white meat may not be obvious in association with CVD prognosis. Essentially when compared to red meat (FAO, 2017), a comparatively smaller percentage of white meat intake is not clearly associated with CVD‐associated substances. Additionally, white meat from poultry is largely cooked and consumed with meat on bones, for example, drumsticks and wings making it difficult for research subjects to recall with certainty the volume of white meat they consume. The potential for data collection bias in studies of CVD prognosis and white meat consumption may have been inconsistent in previous studies. This concern may be addressed by considering total meat consumption meaning data collection bias becomes tolerable and more correlated to CVD (Bernstein et al., 2012; Kim et al., 2017; Larsson et al., 2011a, 2011b).

There is a special interest in comparing CVD mortality and incidence rates between high‐income countries and LMICs. The number of CVD cases and mortality have increased significantly both worldwide and with special regard to LMIC (WHO, 2021). For example, the WHO reported that three‐quarters of CVD deaths occurred in LMIC (WHO, 2021). Our study suggests that the effects of total meat intake were significantly more harmful on CVD incidence in LMIC than in high‐income countries (*z* = 2.52 and 3.49 in Pearson's *r* and nonparametric analyses, respectively, *p* < .010). There may be a couple of reasons which competed with total meat consumption for contributing to CVD incidence making total meat appear “less harmful” in LMIC: (1) This may be attributable to several unique characteristics of LMIC, such as a faster increase of obesity prevalence, rapid urbanization, and emerging socioeconomic transitions to processed foods. All these characteristics have been enabling populations in LMIC to have increasing access to total meat (flesh) intake which led to CVD incidence increase. This may have been reflected in the statistical relationship between total meat intake and CVD (*r* range: .555–.767, moderate to strong correlation, *p* < .001, Table 1). (2) There is more red meat intake in high‐income countries, which should make the correlation between total meat intake and CVD incidence stronger due to higher levels of CVD risk‐associated substances in red meat (saturated fat, l‐carnitine, TMAO, sodium). However, people in higher income countries tend to consume unhealthier diets which include more sugar (*r* = .727, *p* < .001) (You & Henneberg, 2016b) and gluten (*r* = .625, *p* < .001) (You et al., 2020; You & Henneberg, 2016c), but less vegetables and fruits (Kalmpourtzidou et al., 2020). For example, the American Heart Association reported that unhealthy diets in US0A have been contributing to the rise of CVD incidence based noting that 91.9% of Americans have an unhealthy diet, 8% consume a somewhat healthy diet and only 0.1% consume a healthy diet (Mozaffarian et al., 2015). Furthermore, people in high‐income countries tend to be sedentary (less physical exercise) and this has been postulated as a risk factor for CVD pathogenesis (Basu et al., 2013; Mozaffarian et al., 2015).

It is important to consider all aspects of diet as noted in this study which argues for an analysis of total meat (flesh) supply, instead of singling out individual meats for their role in contributing to CVDs. This approach is more in keeping with the manner in which various types of meats, red and white, are consumed as part of people's diet patterns (Tantamango‐Bartley et al., 2015; You & Henneberg, 2016a). Total meat, as the independent variable, is the total amount of “flesh of animals used for food” (FAO, 2018; Lawrie & Ledward, 2006); therefore, other potential confounding factors, such as processing methods and cooking methods, may not affect the correlation of total meat to CVDs.

### 4.1 Limitation of this study

Firstly, population‐level data were included for calculating the relationship between total meat and CVD incidence. Therefore, while the relationships reported in this study may hold true at the country level, they might not be true at the individual level.

Secondly, the CVD incidence rate was downloaded from the IHME database. There is a risk that the data might not be complete in every country, especially in developing countries where record keeping may be incomplete or there are low levels of formal medical diagnoses. Although we tried to remove this potential bias through statistically controlling for GDP PPP and urbanization, the residue may still remain.

Thirdly, total meat (“flesh of animals”) was included as the predicting variable for examining its role in contributing to CVD incidence. However, the potential confounding factors, such as processing and cooking methods, were not considered for our data analyses.

Fourthly, we could only track the general meat supply in the FAO international food database, instead of actual human consumption. Also, there is no direct measure for food wastage for us to calculate the precise measures of food consumption. Therefore, the data on total meat consumption in this study are an approximation.

## 5 CONCLUSION

Worldwide, total meat (flesh) access may be another significant predictor of CVD especially in low and low‐middle income countries. It is worth investigating more about the role of total meat intake in determining noncommunicable diseases, such as CVDs.

## 6 CONFLICT OF INTEREST STATEMENT

The authors declare that there is no conflict of interest regarding this study.

## ETHICS APPROVAL

The data included in this study carry negligible risk and involve the use of nonidentifiable, preexisting data about human beings. Therefore, this study does not require ethical approval.

## Data Availability

The data sources have been described in detail in Section 2. All the data used in this study were freely downloaded from the United Nations (UN) agencies’ websites. No written consent for participation is applicable.
